# Don’t be deceived: bald-faced lies *are* deceitful assertions

**DOI:** 10.1007/s11229-023-04180-9

**Published:** 2023-05-26

**Authors:** Jakub Rudnicki, Joanna Odrowąż-Sypniewska

**Affiliations:** Faculty of Philosophy, University of Warsaw, Krakowskie Przedmiescie 3, 00-927 Warsaw, Poland

**Keywords:** Assertion, Bald-faced lies, Institution, Intention to deceive, Lies, Speech acts

## Abstract

The traditional conception of lying, according to which to lie is to make an assertion with an intention to deceive the hearer, has recently been put under pressure by the phenomenon of bald-faced lies i.e. utterances that *prima facie* look like lies but because of their blatancy allegedly lack the accompanying intention to deceive. In this paper we propose an intuitive way of reconciling the phenomenon of bald-faced lies with the traditional conception by suggesting that the existing analyses of the phenomenon overlook a non-obvious category of hearers whom the speakers of bald-faced lies intend to deceive. Those hearers are institutions represented by the people involved, such as courts or secret police. We also criticize two recent rival accounts (Jessica Keiser’s and Daniel Harris’s) that attempt to save the traditional conception by saying that some bald-faced lies are not assertions, because they are conventional—rather than illocutionary—speech acts.

## Introduction

The traditional conception of lying, according to which to lie is—roughly—to assert^1^ something one believes to be false with the intention to deceive, has recently been confronted with cases of the so-called bald-faced lies. Bald-faced lies are utterances that intuitively seem to be lies (as the name suggests) but, at the same time, because of how undisguised and blatant they are, appear not to involve the speaker’s intention to deceive the hearer. One could think of the following ways out of this conundrum. First, one can ignore the intuitions to the contrary and treat bald-faced lies as non-lies. To do this one may stick to the traditional conception of lying and accept that bald-faced lies are not really lies either because they do not involve the intention to deceive (see e.g. Dynel, 2011), or because they are not even assertions (e.g. Keiser, 2016; Maitra, 2018; Meibauer, 2014). One of the problems that both these approaches face is the difficulty of explaining why we tend to regard such speech acts (non-assertive and/or not involving the intention to deceive) as morally wrong.^2^ They also fail to provide an explanation of why we intuitively regard bald-faced lies as lies. If lying typically involves the intention to deceive, and bald-faced lies do not involve such an intention, it is mysterious why we are drawn to thinking that they are lies. The other way to approach the problem is to try to find a way to claim convincingly that there is a sense in which bald-faced lies are deceptive after all (Lackey, 2013; Marques, 2020).^3^

In this paper, we’ll advocate for a solution of the latter type. Our aim is to provide a straightforward way of showing how bald-faced lies can naturally be seen as involving the intention to deceive. This will allow us to save the traditional conception of lying, endorse the intuitive judgments to the effect that bald-faced lies are in fact lies and assertions, as well as maintain the standard way of explaining why they are morally wrong. We will also try to point out some deficiencies of two recent rival proposals which are also aimed at retaining the traditional conception of lying: Keiser’s (2016) and Harris’s (2020).

Our view can also be understood as an attempt to explain the experimental results which fairly consistently show that folk participants have a tendency to judge typical cases of bald-faced lies as lies (Arico & Fallis, 2013; Coleman & Kay, 1981; Meibauer, 2016; Rutschmann & Wiegmann, 2017) and as deceptive (Meibauer, 2016; Rutschmann & Wiegmann, 2017).^4^ For instance, Meibauer confronted participants with typical cases of bald-faced lies (including Frankie and Takhlef’s cases described below) and asked them, i.a. whether the protagonists lied and whether they acted deceptively. As Meibauer reports “all target utterances were considered as lies” and all “were considered deceptive” (2016, p. 266).

Our plan is as follows. In Sect. 2 we begin by presenting and discussing some typical examples of bald-faced lies that will prove useful throughout the paper. Next, in Sects. 3 and 4, we shall briefly discuss two competing proposals (Keiser’s and Harris’s) and indicate their problems. Finally, in Sect. 5, we shall offer our solution to the problem that bald-faced lies pose for the traditional conception of lying. The solution rests on the observation that in the cases of bald-faced lies of the type that dominates the literature, and which most of our discussion will focus on, there are some non-obvious addressees or hearers towards which the speaker’s intention to deceive is aimed. These addressees or hearers are not always people but sometimes are institutions such as, say, the secret police, courts, etc. The other type of bald-faced lies, those that do not involve institutions, are categorized as cases of irrational attempts to deceive (and are briefly discussed in Sect. 4 alongside the discussion of Harris’s view).

## Examples of bald-faced lies

Let us start with a brief description of three typical scenarios offered as examples of bald-faced lies. First is the story of a Western journalist, Seierstad, and her local minder in Iraq, Takhlef. Takhlef says “Everything [President Saddam Hussein] did in the past was good and everything he will do in the future is good”. When asked by the journalist how he can be so sure about all this, he replies “I know it as a result of my belief in the party and his leadership”. Seierstad knows perfectly well that Takhlef is lying, and Takhlef knows that she knows it. Sorensen, who has introduced this example to the literature, comments on it further as follows:

Takhlef’s superior, Uday al-Taiy at the Information Ministry, is not stupid. Nor is Uday’s pseudo-subordinate, the intelligence officer Kadim, whose job is to keep an eye on Uday. Everybody realizes that Takhlef’s description of Saddam Hussein’s performance is a lie. Everybody knows Takhlef is lying and everybody knows everyone knows it (Sorensen, 2007, pp. 251–252).

The second example is even simpler and involves a witness testifying in court (in one of the versions, the witness is ‘Frankie Five Angels’ Pentangeli testifying at a Senate hearing, therefore in what’s coming when referring to this case, we will call the witness ‘Frankie’).^5^ Frankie says “The defendant is not guilty”. Everybody present at the trial knows that Frankie knows that the defendant is guilty (in different versions of essentially the same story, it can either be a result of the fact that Frankie has been videotaped as witnessing the crime, or of the defendant’s well-known involvement in the Mob and Frankie’s prior settlements made with the prosecutor and the police) but he, nevertheless, decides at the last moment to lie out of fear (depending on the version, he either insists that he has not witnessed the crime, or that he knows nothing about the existence of the Mob).

The third case, presented originally by Carson (2006, p. 290), involves a university student who is caught red-handed cheating on an exam. The student knows about the unofficial policy of the dean who, out of fear of lawsuits against the department he represents, never upholds charges unless a student confesses. The student says “I didn’t cheat”. Even though the dean knows that our student did cheat, and the student knows that the dean knows that, the student—in order to avoid punishment—insists before the dean that she has not cheated.

All three above scenarios are typical cases of bald-faced lies because they all involve situations in which it is common knowledge between the conversationalists that the speaker is saying something false (i.e. the speaker knows that what she says is false, the hearer(s) knows that the speaker’s utterance is false, the speaker knows that the hearer knows that what she says is false, etc.).^6^ At first glance, given that everybody knows that what the speaker said is false, it seems reasonable to conclude that the speaker has no intention to deceive the hearer(s).

## Keiser’s language-games view

Jessica Keiser (2016) distinguishes among bald-faced lies two very different types. One type groups those bald-faced lies which are assertions (and involve an intention to deceive), while the other type is composed of such bald-faced lies that are not assertions and hence cannot be genuine instances of lies. She assumes an epistemic account of assertion—i.e. such an account that posits “an intention in the speaker to affect the epistemic state of the addressee” (Keiser, 2016, p. 469). Hers is a weak epistemic account for she does not require that the speaker by her assertion intends to induce in the audience a belief that she believes in what she asserted, but merely that the speaker intends that her assertion will provide her audience with a reason to believe that she believes in what was asserted. The traditional definition of lying together with the epistemic account of assertion straightforwardly entails the intention to deceive. If one is intentionally giving their audience a reason to believe that one believes something that one knows to be false, then one is “intentionally worsening their epistemic position with respect to this proposition” (Keiser, 2016, p. 469), and—in effect—is deceiving the hearers. Keiser claims that many bald-faced lies will belong to this group but gives no particular examples.

Keiser is more interested in the other group of bald-faced lies which is also more important from our point of view. To this type belong those bald-faced lies in which, just as in the three examples introduced above, there is (apparently) no intent to deceive. In order to make them fit her framework, Keiser has to argue that such utterances are not genuine instances of assertion. And indeed she claims that they are not illocutionary acts, since they are not moves in a conversation. She adapts Bach and Harnish’s (1979) account of illocutionary acts on which such acts are defined by reflexive communicative intentions and argues on such grounds that, for instance, Frankie does not make an assertion. His speech act is a move in another language game, namely in the courtroom game, and is a mere “mock illocutionary act” (Keiser, 2016, p. 471). The courtroom game originally started as a genuine conversation, and as it became more structured and more conventionalized it became a separate game of its own (Keiser, 2016, p. 472). In this game people perform locutionary acts which are not illocutionary acts: Frankie falsely testifying on the witness stand performs merely a locutionary act of transmitting a certain proposition. His utterance is not an illocutionary act, for “he does not intend his audience to do anything with that [proposition] on the basis of the recognition of his intention” (Keiser, 2016, p. 471).

We see several problems with Keiser’s account. First, the distinction between conversations and other specific language games seems arbitrary and vague.^7^ It is not clear how one is to determine whether a given exchange is a conversation or a different game. Many conversations follow more or less determinate scripts (buying things, asking for directions, asking about one’s health, etc.) So, once we start distinguishing those other games it may turn out that we’ll be left with very few ‘genuine’ conversations.^8^

Secondly, the claim that Frankie wants his audience merely to entertain the proposition that he expressed seems controversial. Frankie clearly wants the court to take his utterance at face value and put it on the record. It is true that he does not want to make *the individual judges* believe in what he said (he knows this to be impossible), but he does want *the court* to accept his utterance as true. We might even say that he wants *the court* to *believe* him (see below, Sect. 5) and certainly wants his statement to be recorded for future use.^9^

Thirdly, saying that utterances like Frankie’s are mere locutionary acts (with possible perlocutionary effects) has some unintuitive consequences. First of all, if it were so, it is not clear whether Frankie could be sentenced for perjury. For instance, according to the UK statutory definition, a person is guilty of perjury if they are lawfully sworn as a witness in a judicial proceeding and willfully make a statement material in that proceeding that they know to be false or do not believe to be true (Perjury Act 1911). It is widely accepted that “making a statement” here means roughly the same as “asserting”. For instance, Skoczeń and Smywiński-Pohl note that “for perjury to occur (…) the speaker needs to commit herself to her statement; in other words, he or she must perform the *speech act of assertion*” (2022, p. 327, our emphasis), whereas regarding the similar US statutory definition, Solan writes: “The federal perjury statute criminalizes *an assertion* of a material fact that the speaker believes to be false, but which is *asserted* as true” (Solan, 2022, p. 382, our emphases). Thus, since on Keiser’s view Frankie does not make an assertion, it is not obvious that he could be charged with perjury.^10^

Additionally, one might notice that Keiser’s claim that such utterances are not assertions is even harder to justify on competing approaches to assertion. If one defines assertion in terms of commitments or adding content to the common ground rather than in terms of reflexive communicative intentions, then it becomes even less clear why Frankie’s utterance is supposed not to qualify as an assertion. For instance, it seems uncontroversial that Frankie would be committed to defending his claim if challenged and would be held accountable for what he said, which are characteristic features of assertions on the commitment-based view. Similarly, it is obvious that Frankie wants his statement to be added to the (official) common ground.^11^ So, to sum up: whether or not Frankie’s statement is an assertion seems a moot point even on Keiser’s version of the reflexive-intention view of assertion—which additionally happens to have some well-known drawbacks^12^—and on other accounts, it is even harder to defend.

Keiser considers two alternative strategies for dealing with bald-faced lies and finds them problematic. One strategy is to insist that—contrary to appearances—there is an intention to deceive in all cases of bald-faced lies; the other is to modify the definition of lying and claim that lying does not necessarily involve the intention to deceive. Keiser argues that the problem with the first approach is that one might stipulate that in a given case there is no intention to deceive and “as long as there is still the accompanying intuition that the case is an instance of lying the proponent of the intent-to-deceive condition [on lying] will have a problem to deal with” (2016, p. 463). The problem with the second is that it is unable to distinguish lying from non-literal speech acts (such as “you are the cream in my coffee”).

It’s worth noticing that Keiser’s own account suffers from similar problems. Firstly, on her view, there also are utterances (such as Frankie’s statement) that turn out not to be genuine instances of lies, even though intuitively they are lies. Frankie’s utterance strikes us as a lie, so any view that claims that it is not a lie (for whatever reason) has a problem here. The proponents of the alternative view say that it is not a lie because there is no intent to deceive, Keiser says that it is not a lie because it is not an assertion, but the result is the same: they have to argue that the intuitive view is mistaken.^13^ They have to explain away the intuition that bald-faced lies are lies. Arguably, Keiser’s view—if accepted—does a better job of explaining why bald-faced lies are not lies (not only there is no intent to deceive but also they are not assertions), but just like the rival view, she leaves unexplained the phenomenon that bald-faced lies intuitively seem lies. Any view that claims that in the cases of bald-faced lies there is no intent to deceive should include a kind of error theory explaining why people wrongly think that such utterances are lies.

Against the second view, Keiser argues that it is unable to distinguish lies from certain non-literal speech acts.^14^ Again it seems to us that a somewhat similar objection can be mounted against her own view. In her case, it is bald-faced lies that are hard to distinguish from non-literal speech acts. Compare the ironic utterance of “Jack’s a fine friend” and Takhlef’s utterance of “Everything President Saddam Hussein did in the past was good and everything he will do in the future is good”. According to Keiser, the speaker of “Jack’s a fine friend”, uttered in a situation in which it is clear that what the speaker meant is that Jack is not a fine friend, directly means^15^ that Jack is a fine friend and indirectly means that Jack is not a fine friend. The speaker’s utterance is an indirect assertion, but it is not a direct assertion, because the speaker does not R-intend that her utterance provides a reason to believe that she believes that Jack is a fine friend.^16^ Now take Takhlef’s utterance. On Keiser’s analysis, it is not a direct assertion, for Takhlef does not intend to provide his interlocutor with a reason to believe that he believes that everything President Saddam Hussein did in the past was good and everything he will do in the future is good. However, Keiser suggests that it is at the same time a different (non-illocutionary) act in a different (non-conversational) game and an *indirect* assertion (“perhaps communicating their unwillingness to utter a sentence maligning the regime for fear of retribution, as well as communicating their understanding and willingness to accept this attitude in the other” (Keiser, 2016, pp. 472–473)). How is this different from the “Jack’s a fine friend” case? Both are locutionary acts, neither is a direct illocutionary act; both are indirect assertions, neither is a direct assertion. The only difference is that “Jack’s a fine friend” is not a move in a different game, whereas Takhlef’s utterance is claimed to be such a move. However, as we’ve already argued, in many cases it may be difficult to assess whether there is another—non-conversational—game going on. Therefore, it seems that on Keiser’s account, the difference between bald-faced lies and non-literal speech acts is rather elusive.

As we see it, the main source of this problem is that Keiser does not provide a proper analysis of bald-faced lies. She assumes that we somehow (intuitively?) recognize bald-faced lies and tells us that those that do not involve an intention to deceive should be regarded as conventional rather than conversational speech acts. So that every time we encounter a ‘lie’ that does not involve an intention to deceive, we should conclude that it is not an illocutionary act and look for a different, conventional, game of which it is a part. This however falls short of telling us what bald-faced lies are and how to recognize them. Imagine for instance that the ironic utterance “Jack’s a fine friend” is a part of the testimony, hence a part of the courtroom game, and it is made in a situation in which everyone in the room knows that the speaker means that Jack is *not* a fine friend (and the speaker knows that they know that). Would his utterance be a bald-faced lie then? Intuitively not, but it is false, it is a move in a conventional game, and there is no R-intention to give the audience a reason to believe that the speaker believes that Jack is a fine friend, so it satisfies all of the elements present in the rudimentary characteristic of bald-faced lies of this type provided by Keiser.

## Harris’s intentionalism

Another account that divides bald-faced lies into those that are assertions and those that aren’t is Daniel Harris’s intentionalism (2020). Harris argues that bald-faced lies form at least three different categories: (i) irrational attempts to deceive, (ii) indirect speech acts, and (iii) conventional acts. (i) comprises assertions with intent to deceive, (ii)—assertions with no intent to deceive, and (iii)—acts that are not assertions. We agree with Harris about his group (i): some bald-faced lies are irrational attempts to deceive. In many cases, such lies will be a result of the speaker momentarily and irrationally coming to believe that they will manage to deceive the audience (even though the situation is such that it is impossible) (see Harris, 2020, p. 10, fn. 13). The other two groups that Harris identifies are more problematic. Let’s discuss them in turn.

Harris argues that some bald-faced lies are “indirect speech acts, wherein the speaker makes as if to assert something in order to indirectly accomplish some other conversational goal” (2020, p. 11).^17^ This resembles Keiser’s remarks concerning indirect assertions, but the crucial difference is that while Keiser uses a weak non-Gricean notion of *meaning something* (see above), Harris appeals to the traditional Gricean one. As we have seen, Keiser says that the speaker of an ironic utterance “Jack’s a fine friend” directly means that Jack is a fine friend (Keiser, 2016, p. 466), whereas for Harris such a speaker would not mean that at all, and hence would only make as if to say this. Thus, bald-faced lies in group (ii) will be cases in which the speaker merely makes as if to say something in order to indirectly communicate something else. As in Keiser’s theory, the message conveyed indirectly amounts to the speech act’s implicature.

Harris gives an example of a husband saying to his wife “I slept in my office last night” when it is obvious to both of them that he spent the night with another woman.^18^ According to Harris’s interpretation, if the husband doesn’t irrationally intend to deceive his wife, then the point of his utterance is to communicate that he feels no need to apologize (Harris, 2020, p. 12). This account likens bald-faced lies to non-literal speech acts, such as irony and metaphor. The problem with this is that we do not usually treat ironic or metaphorical utterances as lies.^19^ Harris, however, seems to imply that bald-faced lies in this group just *are* non-literal speech acts. The reason why Grice says that the speakers of “Jack’s a fine friend” and “You are the cream in my coffee” do not say—but merely make as if to say—that Jack is a fine friend and you are the cream in my coffee, is that they do not *mean* that Jack is a fine friend and you are the cream in my coffee. The problem with Harris’s proposal is that bald-faced liars usually *do* mean what they say. And if they mean what they say, there is no reason to argue that they merely make as if to say what they literally say. Harris gives the following example:

Ann and Bob have divided up the chores. Bob’s job is to mow the lawn at 2 p.m. At 2:05, Bob is still playing a video game. The following exchange unfolds:

Ann: What time is it Bob?

Bob: Oh, it’s about 1:45.

Let’s suppose that it’s completely obvious that it is after 2 p.m., and so Bob can’t be (rationally) attempting to deceive Ann. And let’s suppose that he’s not irrationally attempting to deceive her. In that case, his most plausible real aim is to indirectly inform her that he’s not going to start mowing the lawn quite yet (and perhaps to rub it in her face that she can’t make him). (2020, p. 12).

He also argues that something similar might be said about the unfaithful husband’s utterance “I slept in my office last night”. The way we interpret those examples however is that they are indeed indirect speech acts, but acts in which speakers say (and not merely make as if to say) what they have uttered. We can easily imagine the exchange between Ann and Bob to continue as follows:

Ann: You know that’s not true. It’s already past 2 pm.

The wife could reply in a similar way (e.g. “We both know that’s not true”). The fact that Ann and the wife can naturally reply in this way suggests that they take what was uttered as asserted. The situation with indirect speech acts in which speakers merely make as if to say is different. If Ann said sarcastically “Jack is a fine friend” and Bob replied “That’s not true, he’s a terrible friend”, that would be evidence that Bob *misunderstood* Ann’s utterance.

On the other hand, if Harris gave up on the claim that in bald-faced lies one only makes as if to say, then we would be left with the claim that bald-faced lies are indirect speech acts in which the speaker indirectly communicates something other than the literal content of the utterance, which does not distinguish bald-faced lies from other speech acts.

Bald-faced lies that belong to group (iii) are—according to Harris—conventional rather than communicative acts. He suggests that both Frankie’s legal testimony and the cheating student’s utterance are best seen as conventional acts. He—like Keiser—argues that Frankie’s utterance is not an assertion, but—unlike Keiser—claims that it is a lie nonetheless.^20^

According to Bach and Harnish (1979), conventional acts are institutional and do not require R-intentions:

Communicative illocutionary acts succeed by means of recognition of intention, whereas conventional ones succeed by satisfying a convention (Bach & Harnish, 1979, p. 110).

Bach and Harnish claim that “For an utterance to be a conventional illocutionary act, not only must it be the utterance of what the convention requires (the specified words, or words with the specified meanings), it must be issued by the right person under the right circumstances” (1979, p. 110).

While it seems plausible that finding a defendant guilty is a conventional rather than communicative act (it must be done in an appropriate manner, by an appropriate person, at an appropriate moment of the proceedings), the same cannot be said about testifying at the witness stand. Speaking under oath does not deprive the utterances of their assertoric status. Witnesses can be challenged; their testimony can be doubted or confirmed. Unlike judges’ verdicts, statements of the witnesses do not necessarily have a normative impact. The role of the witness is different from that of the judge. Witnesses are called to testify what they know about the case, and the judges and the jury members are interested in their knowledge, hence in the content of utterances that they make. The fact that testifying under oath might be regarded as an institution, a conventionalized procedure, does not amount to the content of the evidence given being conventional rather than communicative.

Thus, while we agree with Harris that “[t]he very possibility of testifying in court depends on an elaborate system of institutions and procedural conventions that vary by jurisdiction” (Harris, 2020, p. 15), we do not accept that what is said during the testimony is not an assertion. Harris—like Keiser—argues that the aim of sharing information or influencing people’s beliefs is at best secondary in legal testimony. He invites us to compare such testimony to the situation in which the witness asserts to the same audience everything that he is about to testify but he does so on the steps of the courthouse. Harris claims that although the noises he makes in front of the courtroom are similar (or even identical) to the noises he makes during his testimony, only the former are assertions, and the latter are “fundamentally different speech acts” (Harris, 2020, p. 15). It seems to us however that a more plausible view is that it is the same type of speech act made to a different audience. Frankie repeats his assertions in front of the court (the institution) whereas previously he made them in front of the people who happen to be members of the court (a group of individuals). For conventional reasons, testifying in front of the court is different from talking to the members of the court in their free time, but that doesn’t mean that only the latter involves assertions. In our opinion, it is better to regard acts like legal testimony as involving assertions, their partial conventionalization notwithstanding. Plausibly such acts are not merely assertions but other acts as well. Compare the situation in which someone says “It’s very hot here”. Let’s assume the speaker knows that everybody knows that it is hot here, so she cannot intend to share this information and change people’s beliefs. She’s hoping however that her utterance will be understood as a request to open the window. That doesn’t mean that her utterance is *only* a request: on the contrary, it is an indirect speech act in which she makes a request by means of making an assertion. The fact that her utterance is primarily a different (indirect) speech act is not proof that it is not also a (direct) assertion. It might be an infelicitous assertion (because it states what is obvious in this situation), but an infelicitous assertion is still an assertion. Similarly here: although transmitting information is not Frankie’s main goal, he’s still asserting that the defendant is not guilty.

As far as the cheating student is concerned, Harris argues that if the student’s utterance is considered an assertion, it has to be seen—counterintuitively—as a failure, since the student didn’t convince the dean that she didn’t cheat. However, her speech act clearly is not a failure—her aim wasn’t to convince the dean that what she says is true (she didn’t want the dean to treat it as true for the purposes of the conversation), but rather she wanted the dean to accept it for “official disciplinary purposes” (Harris, 2020, p. 6) which is an “extra-conversational matter” (Harris, 2020, p. 5). We agree that the student cares about the official disciplinary purposes and not about the purposes of the conversation, but we do not think that this results in her utterance not being an assertion (see below, Sect. 5). Again, it might well be the case that her speech act is both an assertion and some other speech act. The fact that it does not succeed as an assertion towards the dean doesn’t mean that the whole speech act is a failure. To motivate his solution, Harris notices that the conversation between the student and the dean could continue as follows:

STUDENT: I didn’t cheat.

DEAN: That’s false and you and I both know it. But I am not going to punish you since you haven’t confessed. You’ll just have to live with your guilt. (2020, p. 6).

*Pace* Harris, it seems to us that the dean’s answer may be taken as a reason for treating the student’s utterance as an assertion as well as some other act. After all, the dean says “That’s false”. His reaction suggests that he does regard the student’s claim as a normal assertion. He is of course aware that it is to serve some other purpose as well, but that does not mean that it is not an assertion.^21^

## Our solution

In the preceding sections, we have pointed out several weaknesses of the two accounts that try to uphold the traditional definition of lying by arguing i.a. that some bald-faced lies are not assertions. It is now time to present our own solution, which is meant to be a defense of the view that all bald-faced lies are assertions whose speakers intend to deceive their audience.

Let’s begin the presentation of our solution by taking a closer look at the traditional definition of lying stemming from Augustine’s remark that “In reality, the fault of a person who tells a lie consists in his desire to deceive in expressing his thought.” (1952 [395], pp. 55–56). This remark has led to the formulation of the following definition of lying centered around the Augustinian suggestion of the centrality of deception:

A lies to B if and only if (I) A expresses *p* to B, (II) A believes that *p* is false, and (III) by expressing *p*, A intends to deceive B into believing *p*.

There is an immediate problem with this definition, which has been made evident by recent scale-effect objections. Roy Sorensen (2022) argues that focusing on “small scale” scenarios, consisting of one speaker and one addressee, makes us blind to the fact that conversational situations in which lies are uttered are sometimes much more complex. Among the cases that he discusses is the example of eavesdroppers, which is “a counterexample to the requirement that the liar intends to deceive the *addressee*” (2022, p. 130, his emphasis). In Sorensen’s scenario, during World War II, the UK Ministry of Food intended to deceive German eavesdroppers rather than the British people with their announcements that carrots improve night vision. If we rely on the above definition, we’ll have to conclude that the Ministry of Food didn’t lie. They expressed a false proposition to the British people but had no intention of deceiving them. They intended to deceive potential German eavesdroppers, who, however, were arguably not the addressees of the announcement. This verdict appears incorrect, for it seems that by making the claim that carrots improve night vision, which they didn’t believe, the Ministry of Food did lie. A similar problem arises for undisguised lies (of which bald-faced lies are a subclass). Imagine a politician telling an undisguised lie when addressing the voters. To the politician, it is clear that most of the hearers know perfectly well that he is lying, and therefore, he can be said not to intend to deceive those. At the same time, it is reasonable to assume that he believes that there are at least some vulnerable people among those whom he is addressing, who can be deceived. According to the above definition, we would have to conclude that the politician is lying only to the people belonging to the latter group but not to those belonging to the former. This seems wrong for, intuitively, he is lying (lying simpliciter) if he wants to deceive anyone at all. This motivates the following minor change in the traditional definition:“A intends to deceive at least one hearer of A’s utterance expressing *p* into believing *p*” is meant to be read *de dicto*, i.e. as: A intends that there is a hearer such that A deceives him (and not as: there is a hearer such that A intends to deceive him).

A lies if and only if (I) A expresses *p*, (II) A believes that *p* is false, and (III) A intends to deceive at least one hearer of A’s utterance expressing *p* into believing *p*.^22^

This modification falls in line with the more general natural intuition that one can lie (*simpliciter*) to a wide, unspecified audience; for example by making a deceitful public statement, via a public report, or in a television commercial (Mahon, 2015).

The revised definition does not allude in any way to the idea of an addressee, or a primary interlocutor, understood as the direct—or directly intended by the speaker—receiver of the expressed proposition (contrary to the standard version that involves a particular receiver, B, who is the direct, deliberate addressee of A’s expression of *p*, and the one A is trying to deceive). So for example, if our politician’s utterance is an answer to a question posed during an interview with a journalist, as long as the answer is aimed by the politician also at a wider audience, we do not need to implausibly restrict the set of hearers only to the journalist. As a result, we are also able to accommodate the fact that the politician does not need to have anyone particular in mind as the receiver(s) and as the one(s) to be deceived by his lie. Also, it might happen that every person that hears his utterance belongs to the group of those who know perfectly well that the politician is lying and nobody will be deceived. In such a case, we would still like to claim that he lied and the reason for that is precisely that he intended to deceive *someone*. The fact that he was mistaken in assuming the existence of vulnerable voters should not matter. In other words, even if it was impossible to deceive any of the actual hearers, the utterance still counts as a lie because the speaker wanted to reach some people who could potentially be deceived and they were the ones he can be said to have intended to deceive.

The suggested modification of the traditional definition of lying makes room for our proposal. The crux of it is that when it comes to the typical analysis of examples of bald-faced lies, the analysis overlooks some hearers involved in those cases. In each story, there are hearers that are not mentioned explicitly in the description of the examples, but which make all the difference. The most straightforward take on who the hearers are in the three scenarios is that in the first it is the journalist, Seierstad, as well as Takhlef’s superior, Uday, and probably Uday’s pseudo-subordinate, Kadim; in the second case, it is the prosecutor, the judge, the jurors, and other people present during the trial; and in the third case, it is the dean. However, our suggestion is that these lists are incomplete. We think that the three stories are best analyzed as involving additional hearers whom the speaker in fact wants to deceive by lying.

In the first case, it is the Information Ministry and the intelligence agency as institutions. It is true that Takhlef does not intend to deceive the mentioned employees of those institutions since they know that Takhlef is lying. At the same time, the system is organized in such a way that they are forced to place it on record and alarm their institutions once Takhlef says something that would stand out from the standard dictator-loving script. It is in this sense that Takhlef not only intends to deceive the two institutions but is in fact successful. Note also that, just as in the case of our politician, it does not matter whether Takhlef knows for sure that his utterance is listened to by those institutions or even whether it is in fact listened to or not. What is relevant is that he has the intention to deceive the institutions to be on the safe side *in case* they were listening.

The second case is such that it is the court that is the additional hearer. Even though we can assume that the judge and the jurors know that the witness is lying, the legal system is organized in such a way that this is not enough for the defendant to be convicted. In other words, the court as an institution may become deceived even if no person involved in the trial is. This is exactly the effect the witness is trying to achieve. He has no intention to deceive the invulnerable people present but he does intend to deceive the court as an institution which can result in the defendant being freed—the outcome the witness hopes for in order to remain safe.

The same strategy should be used to analyze the third scenario. Here the obvious hearer is the dean and the conversation would be normally understood as taking place between him and the cheating student. As was already discussed, the student knows that the dean knows that the student cheated, and therefore, the student has no intention to deceive the dean understood as the human before the student’s eyes. At the same time, just as in the previous examples, the dean is only a member or a representative of the institution he works for—the university department, which, according to us, should be seen as a hearer in this story too. After all, just as it is not the judge as a person but the court as an institution that the judge represents that makes decisions with regard to the defendant’s guilt, it is not the dean as a person but the department that decides whether to punish cheating students or not. It is this very institution that is supposed to decide whether the student should be punished or not, and the private opinion of the dean as a person does not need to cohere with the institution’s official verdict. And this is exactly what happens in the scenario once the student insists that she has not cheated. She, knowing about the unofficial policy, intends to deceive the institution, even though she does not intend to deceive the dean-person. There is a clear difference between the dean and the department. The unofficial policy regulates what the department will do but it does not affect what the dean as a person thinks about the particular case. The policy makes it possible for any student to deceive the institution because of how it is formulated—analogously to how the rules of trials as well as of the surveillance institutions of totalitarian regimes are codified—regardless of whether the student believes the dean can be deceived or not.^23^

In this respect, our solution resembles that of Harris. As we have seen, he argues that the student wants the dean to accept her utterance as true for official disciplinary purposes (i.e. on behalf of the department) and doesn’t care whether the dean regards it as true for the purpose of the conversation (2020, p. 6). However, the price that Harris has to pay is the denial that the student’s utterance is an assertion. Our approach allows us to ‘have the cake and eat it too’: by distinguishing the two hearers, the dean and the department, and by noticing that the student’s attempt at deception was directed towards the latter, we don’t have to give up on the claim that her utterance is an assertion. Contrary to what Harris suggests (see above), even seen as an assertion, the student’s act doesn’t have to be seen as a failure: the student clearly achieved her aim, since she succeeded in deceiving the department.^24^

These observations together with the straightforward and otherwise plausible modification of the typical formulation of the traditional definition of lying solve the problem posed by bald-faced lies. Once we have identified the overlooked hearers of the relevant utterances, bald-faced lies can be analyzed in a way analogous to how standard lies are, i.e. as assertions aimed at deceiving. The only difference in this regard is that for the class of bald-faced lies that gathered the most attention in the literature, i.e. for those exemplified by our three main scenarios, the aim of the deceiving is targeted at a somewhat non-standard hearer—an institution such as a court, an intelligence agency, or an academic institution.^25^^,^^26^

One might object that ‘institutional hearers’ do not satisfy our version of the traditional definition of lying, for it is not possible to deceive an institution *into believing p*. However, it is now common in social epistemology to ascribe beliefs to groups, collections of people, or institutions,^27^ and we take the idea of an institution believing that *p* to be grounded in the everyday linguistic practice of saying things like: “The CIA believes North Korea’s missile program is aimed at coercion…,” “Police believe a suspect in the killing of a woman who was found dead over the weekend in Ames, Iowa, is in Kansas City.”, “Biden administration believes WHO is vital to containing Covid-19 pandemic.”^28^ Furthermore, we also share Tollefsen’s (2002) analysis of the nature of the mentioned practice as not merely metaphorical or semantically reducible to speaking about the attitudes of the members of the institutions in question,^29^ and as stemming from the interpretationist approach towards intentionality. According to this view, inspired by the work of Dennett (1981, 1987), ascriptions of intentionality and of attitudes (such as belief) to an entity, follow naturally from the assumption of its rationality.^30^ And this assumption has to be in place if we want to make sense of the entity’s decisions and actions.

And it seems clear that when it comes to typical institutions, including courts, intelligence agencies, and university departments, we do in fact want to be able to explain their decisions, predict their actions, and so on, especially given that we know that they have been designed and founded in order to be able to undergo such rational procedures aimed at fulfilling certain goals as a result of processing appropriate input.^31^^,^^32^

So, for example, in our cheating-student case, and restricting the scope of the department’s actions and interests only to cases of this sort, the rational-intentional structure of the department can be analyzed as follows. Its main goal is to avoid lawsuits, i.e. to believe the students to be guilty and behave as if they were only if they confess, and believe them not guilty and behave as if they were so otherwise. Its means to achieve this goal, i.e. its subgoal, is to correctly identify whether a student accused of cheating confesses or not. And this identification is commissioned to the dean who is supposed to talk to the accused students and form a belief as to whether they confessed or not. Note that this rational decision structure aimed at forming the department’s belief with regard to the guilt of the students extends beyond the dean (i.e. the individual(s) involved) in two relevant senses. First, his judgment of whether the students confess or not is only an input into the more general decision process of the department aimed at producing the department’s belief about the guilt of the students. And second, the dean’s personal belief with regard to the students’ guilt is, within the structure designed the way it is, completely irrelevant to the formation of the department’s belief about this matter.

The above should not only motivate the general treatment of the institutions we are interested here in as belief holders and agents but also shows that they are treated as such by the speakers in our stories, i.e. Takhlef, Frankie, and the cheating student. That is because their utterances aimed at deceiving the relevant institutions need to take into consideration, at least roughly, the rational decision- and belief-forming procedures that those institutions operate under. For example, if the cheating student didn’t assume the rationality of the department in its execution of some belief-forming procedure processing a non-confession into a belief about the student’s innocence, she could not reliably expect to get away with cheating after not confessing during the conversation with the dean.

For all the above reasons, it might be argued that our initial intuitive judgment was correct: bald-faced lies are lies. Some—like the case of the obviously drunk man insisting that he hasn’t been drinking (cf. Harris, 2020, p. 10) or of the gambler denying to his wife that he had been to the racetrack directly after she pulled betting tickets out of the pocket of his coat (Arico & Fallis, 2013, p. 814)—are irrational attempts to deceive fellow human beings, whereas some—like the courtroom case—are perfectly rational attempts at deceiving relevant institutions. As such they are usually worthy of moral condemnation.

One might argue, though, that deceiving institutions might be less condemnable than deceiving people. When you deceive a person, you often harm (or at least risk harming) them: if they act on the basis of your lie, they can’t “effectively pursue [their] ends and interests” (Carson, 2018, p. 147), and harming others is (in typical cases) uncontroversially wrong. Harming institutions in this way might seem less reprehensible. However, when one lies to an institution, who ends up being harmed as a result are also people. Such a lie will very likely impact the behavior of the institution in a way that prohibits it from effectively realizing the ends and interests of the people served by it.

Whereas we think that all bald-faced lies involve an intention to deceive, we do not think that all cases in which someone utters a blatant falsehood should be classified as bald-faced lies. It seems to us that examples in which there is absolutely no one who is meant to be deceived (be it a person or an institution), do not qualify as lies, even if they involve saying something false. We think that the reason why the typical cases that we’ve discussed are classified as lies is that in those cases there are agents that are being deceived. If there are no such agents, then the intuitions that we have to do with lies are much weaker.^33^

## Conclusions

In this paper, we have argued that the phenomenon of bald-faced lies does not force us to reject the traditional conception of lying by restoring the idea that bald-faced lies are assertions made with an attempt of deceiving. We achieved this result by noticing that the typical analysis of the standard cases of bald-faced lies, i.e. those allegedly unaccompanied by the intention to deceive, is incomplete with regards to the hearers it allows to treat as the targets of the speaker’s intention to deceive. Once a broader look that we advocate for is taken, it becomes clear that in all of those cases there, in fact, are hearers of an institutional type involved, and it is those institutions that the speakers are trying to deceive.

It is perhaps worth stressing again that we do not claim that *all* bald-faced lies are attempts at deceiving institutions. There are bald-faced lies that are irrational (often desperate) attempts at deceiving typical hearers. When a thief is caught with his loot and claims that it wasn’t stolen, it’s quite probable that he hopes (irrationally of course) that the police will somehow believe him.

Along the way, we have criticized two competing proposals, Keiser’s (2016) and Harris’s (2020), aimed at saving the traditional conception by categorizing at least some bald-faced lies as non-assertions. We’ve tried to argue that their claims to that effect are not convincing and have some problematic consequences. In our opinion, such a move can be avoided if one broadens the category of hearers or the audience so that it includes not only people but also institutions (represented by those people). Given the problems of the mentioned proposals, we believe that we have made a stronger case for restoring the traditional conception of lying as well as treating bald-faced lies as assertions.

By arguing that bald-faced lies—contrary to appearances—involve intentions to deceive, we have argued for the traditional definition of lying. One might object however^34^ that our efforts are wasted since bald-faced lies are not the only example in the repertoire of non-deceptionists, who claim that e.g. knowledge-lies, indifferent-lies, coercion-lies also might not involve intentions to deceive. Nevertheless, bald-faced lies are often regarded as “[the] main argument to prove that intention to deceive is not a necessary condition for lying” ((Wiegmann et al., 2017, p. 603). As Ishani Maitra remarks, there is a “general consensus in the recent literature” that bald-faced lies “require us to abandon the intent-to-deceive tradition” (2018, p. 65). Our analysis may therefore be seen as a significant weakening of the non-deceptionists’ case. Moreover, our solution might be helpful in dealing with some of the other types of purported non-deceptive lies as well. As we’ve already mentioned, our modified definition of lying allows dealing with Sorensen’s German eavesdroppers case. In our definition, we avoid mentioning the addressee and talk about the hearer instead. Our condition III says that A lies if A intends that there is a hearer such that A deceives him, where the hearer doesn’t need to be a direct, intended addressee of the lie. Hence, on our account, the intended eavesdroppers (Germans) straightforwardly count as hearers to whom the speaker might lie. Another case that Sorensen (2022) discusses is “a single lie from shifting perspectives” (130).^35^ A boy says “Santa gave me a beach ball”. He doesn’t lie to his twin sister who informed him earlier that Santa doesn’t exist,^36^ but he’s trying to deceive his younger brother, who still believes in Santa Claus. On our account, there is no doubt that the boy lies because there is a hearer that he wants to deceive (namely his brother). We do not claim that our solution helps to deal with all the problematic cases, but we remain optimistic that if those examples are analyzed case by case, it will turn out that either they’re not lies or they do involve an intention to deceive after all.

## Footnotes

1 There is a strand in the recent literature on lying according to which lies are not restricted to assertions. For example, Viebahn (2020) argues that one can also lie via presuppositions and Marsili (2021) offers a definition of lying involving the notion of assertoric commitment which he understands as related not only to assertions but also to a class of explicitly performative promises and oaths [which on some accounts are thought of as “illocutionarily entailing” assertions (Searle & Vanderveken, 1985)]. We remain open to the mentioned possibility but have decided to frame the discussion in the current paper around the dominant view which treats asserting as necessary for lying. This is further motivated by the fact that a lot of the discussion of the topic of bald-faced lies assumes the dialectical significance of the assertion condition on lying (e.g. Harris, 2020; Keiser, 2016; Maitra, 2018; Marques, 2020; and Meibauer, 2014 are all voices on the topic of whether bald-faced lies constitute assertions). Finally, the main topic of our paper is whether bald-faced lies can be squared with the intention-to-deceive condition on lying. Our argument to this effect remains relevant even if one substitutes the assertion condition with e.g. Marsili’s assertoric-commitment condition.

2 This is not to say that all instances of bald-faced lies necessarily need to be morally wrong. We remain open to the possibility of the existence of the bald-faced equivalents of e.g. white or prosocial lies. The thought here is rather that a good theory of bald-faced lies needs to be able to explain the intuitive moral wrongness of those typical cases of bald-faced lies that are, in fact, morally wrong, just as much as a good theory of lies (sensu *largo*) needs to be able to explain the intuitive moral wrongness of typical cases of lies other than of the white or prosocial kind. Also, we don’t want to be understood as suggesting that it is *impossible* to explain the moral wrongness of bald-faced lies without treating them as assertions aimed at deceiving (for instance, Meibauer (2014) explains the phenomenon by treating certain cases of bald-faced lies as non-assertoric insults). Rather, the claim here is simply that if one denies the assertoric and deceitful character of bald-faced lies, one thereby rejects the most straightforward explanation of their moral status and needs to present an alternative. We would like to thank an anonymous referee for urging us to clarify this issue.

3 Another possible reaction is to accept the difficulty of the challenge posed by bald-faced lies and drop the deception requirement from the definition of lying (Carson, 2006; Fallis, 2010; Saul, 2012; Sorensen, 2007; Stokke, 2013).

4 One exception to this general tendency are the results obtained by Krstić and Wiegmann (2022) for what they call “tell-tale sign bald-faced lies”, i.e. utterances (1) whose literal content is false, (2) their falsity is common knowledge due to a tell-tale sign such as the speaker’s blushing, and (3) whose speakers intend the hearers to deduce the true negation of the literal content based on the tell-tale sign signaling the falsity of that content. Such utterances were judged by participants to be lies but not to be intended by the speakers to deceive. The natural worry regarding the mentioned scenarios, though, is that there seem to be good theoretical grounds to doubt whether they, in fact, constitute lies. The reason is that the content that the characters from the scenarios in fact want to communicate, contrary to the one they literally express, is believed by them to be true. This likens these cases to others not conducive to lying, such as those of irony, where the literal, believed to be false, content is merely made as if to say (Grice, 1989, p. 34). One possible explanation for why the participants nevertheless judged them as lies is that they perhaps interpreted the question as concerned with whether the literal content is believed by the speaker to be false.

5 See e.g. Carson (2006), Keiser (2016).

6 We do not mean that one cannot classify as bald-faced lies cases in which it is not common knowledge that the speaker is saying something false but in which, nevertheless, the hearer does know that the speaker is saying something false, and the speaker is irrational in believing that she can deceive the hearer (like the case of the obviously drunk man insisting that he hasn’t been drinking [cf. Harris, 2020, p. 10)]. In fact, as will be discussed in Sect. 4, we analyze such cases as involving an irrational intention to deceive the (non-institutional) hearer, i.e. differently from how we approach the cases of bald-faced lies introduced above, which we treat as accompanied with an intention to deceive institutions (see Sect. 5). Whether cases of this sort should, in fact, be treated as instances of bald-faced lies seems to us a purely terminological issue, though. If a positive answer to this question is assumed, our analysis of the phenomenon should be understood as bipartite. If a negative one is assumed, our analysis of bald-faced lies is constrained to the institutional element only. For the rest of the paper, we shall assume the former possibility, but, at the same time, we shall devote most of our discussion to the cases presented above that we analyze as involving intentions to deceive institutions. Those cases are the ones that are the most characteristic of the literature on bald-faced lies. We would like to thank two anonymous referees for pressing us to clarify this issue.

7 Another example of such a game that Keiser mentions is the situation of an exam.

8 Similar concerns are voiced by Marques (2020). Marques also notices that while we do accuse bald-faced liars of lying, we do not make that accusation against those who make non-assertions (actors performing on stage, those who make wild conjectures, etc.), which suggests that bald-faced lies—like other lies—are assertions (Marques, 2020, p. 7).

9 In “The Godfather Part II” movie the senators take Frankie’s denial to contradict his earlier sworn statements. It seems that in order to think that Frankie contradicts himself, they must take his utterance as having illocutionary force.

10 Moreover, since in the Canadian Criminal Code “intent to mislead” is necessary for perjury (see e.g., http://criminalnotebook.ca/index.php/Perjury_(Offence)), Frankie, as allegedly only wanting the audience to merely entertain his expressed content, would have nothing to worry about there.

11 See Stokke (2018).

12 See e.g. MacFarlane (2011), Marsili (2023a), Siebel (2003, 2020).

13 Harris admits that “it is unintuitive, or contrary to the way that we would ordinarily use the verb, ‘to lie’, to say that bald-faced lies aren’t lies” (2020, p. 7), but goes on to argue that such an intuition doesn’t carry much weight. We disagree. We think that the notion of lying is different in this respect from the notion of assertion, which indeed “is a theoretical posit that earns its keep by playing a role in a theory of human communication” (2020, p. 7). Lying is a concept deeply ingrained in ordinary conversations and one would need a really good reason to depart from folk intuitions connected with this notion. No such reason has been offered yet, so we are after a descriptive definition of lying, which reflects the ordinary understanding, rather than a revisionary one (see Fallis, 2010; Marsili, 2023a).

14 Keiser’s criticism is directed against Stokke’s version of the common-ground view of assertion. In his reply Stokke argues that her objections are unfounded (Stokke, 2017).

15 Keiser defines direct meaning as a locutionary act of intending for the audience to attend to the proposition expressed in the utterance (see 2016, p. 466).

16 Keiser defines indirect assertions as follows: “Indirect Assertion: By uttering σ, U indirectly asserted *p* iff for some audience A, 1. By uttering σ, U indirectly meant *p.* 2. By uttering σ, U asserted *p*”. while indirect meaning is defined as: “Meaning_I_ (Indirect Meaning): By uttering σ, U meant_I_ *p* iff for some audience A, there is some content *q* (distinct from *p*) such that 1. By uttering σ, U meant *q.* 2. By uttering σ, U meant *p.* 3. U uttered σ R-intending that A recognize (2) at least partially on the basis of (1)” (Keiser, 2016, pp. 467–470).

17 Harris’s understanding of “indirect speech acts” is different from that of Searle. Searle’s indirect speech acts are “cases in which one illocutionary speech act is performed indirectly by way of performing another” (1979, p. 31). So irony is not an indirect speech act in Searle’s sense, whereas it is an indirect speech act on Harris’s interpretation. We’d like to thank an anonymous referee for bringing this difference to our attention.

18 The example comes from Meibauer (2014, p. 127).

19 As we have seen, Keiser claims that the inability to distinguish lying and non-literal speech is a serious drawback of any theory of lying.

20 Harris does not offer a definition of lying. It is clear, however, that he rejects the traditional definition.

21 Again, it seems much harder to deny that such utterances are assertions on the approaches which define assertions in terms of commitments or common ground.

22 The intention to deceive condition of the traditional definition is typically formulated as requiring an intention that the other person come to believe the expressed proposition. For the reasons of brevity and given that this issue is not central to the topic of the paper, we shall assume this approach without further discussion even though we acknowledge that there might be good reasons to modify this condition. For example, if one wants to analyze the cases of so-called knowledge-lies (Sorensen, 2010) as involving an intention to meddle with people’s beliefs (and not just knowledge), one should probably modify the intention to deceive in such a way that deceiving is not understood as necessarily requiring that the speaker intend the addressee to adopt a belief in the expressed proposition but rather as requiring that she intend to increase (even ever so slightly) the addressee’s credence in the expressed proposition. Such a strategy with regards to knowledge-lies is adopted by Fallis (2011) and Staffel (2011). We would like to thank an anonymous referee for pressing us on this issue.

23 An anonymous referee asks for our analysis of a case in which country A is invaded by country B and country B’s representatives, in order to show their contempt “for A, its citizens and everybody else”, insist on the bald-faced lie that the citizens of country A have themselves invited country B’s army. We believe that if the bald-faced lie in question is told—as it typically happens in cases of political propaganda—with at least the slightest hope of deceiving anyone, be it some gullible citizens of other countries or some citizens of country B who sympathize with their government, then this is a case of a (possibly irrational) attempt to deceive and constitutes a lie. It is hard to imagine that it could be uttered without any (however irrational) intention to deceive, but if this were so, then it would be an example of an indirect speech act: an insult performed via an assertion.

24 Obviously, our proposal cannot be reconciled with these versions of the traditional definition of lying which explicitly require that it is a *person* who is to be deceived (see e.g. Carson’s L1 definition (2006, p. 287), unless one acknowledges institutional persons (see fn. 30)). We take it to be an additional advantage of our view since such versions can’t accommodate the natural intuition that one can, say, lie to a tax office.

25 The idea of institutional hearers allows also to capture the intuition that in many cases of bald-faced lies the speakers want to “go on record”, for “going on record” might be regarded as addressing such an institutional hearer.

26 Another important difference is that in cases of bald-faced lies—unlike in cases of other lies—the sincerity condition of assertion is *blatantly* violated. We’d like to thank an anonymous referee for this observation.

27 On group belief see e.g. Pettit (2003), Lackey (2018), Marsili (2023b). See also Ludwig (2017), who argues that “to say ‘Corporation X believes that *p*’ is to treat the proposition *p* as one in accordance with the truth of which the corporation will act given its official goals and what else it accepts (in the same sense) as true” (2017, p. 24). We believe that our solution is consistent also with Ludwig’s approach.

28 https://www.reuters.com/article/us-northkorea-missiles-cia/cia-believes-north-korea-weapons-aimed-at-coercion-not-just-defense-pompeo-idUSKBN1FC2EZ?il=0. https://www.msn.com/en-us/news/crime/police-believe-a-suspect-in-the-killing-of-a-woman-in-ames-iowa-is-in-kansas-city/ar-BB1dWRhH. https://www.hindustantimes.com/world-news/biden-administration-believes-who-is-vital-to-containing-covid-19-pandemic-kamala-harris-tells-who-chief-101611318797392.html.

29 One classic argument against the possibility of reducing the beliefs of institutions to the beliefs of their members is the so-called discursive dilemma (Pettit, 2003), i.e. a situation of inconsistency in institutional decision-making in which two equally good preference-aggregation procedures lead to contradictory results depending on which set of propositions is voted on.

30 A similar view is defended by Philip Pettit (2003), who argues that integrated groups of people can constitute institutional persons, which just like natural persons, are committed to rational unity, i.e. they “have intentional states and perform associated actions in a way that satisfies rational unity” (2003, p. 184) and they acknowledge those states and actions as their own and take responsibility for them (185). Pettit argues that social integrates are distinct from and not reducible to its members, but stresses that he’s not “postulating any ontological mystery” and the institutional persons “do not constitute an ontologically emergent realm” (2003, p. 184). He also notices the differences between natural persons and institutional persons. The latter “are not centres of perception or memory or sentience, or even of degrees of belief and desire”, “form their collective minds only on a restricted range of matters” and do not reason in such a spontaneous way as humans (2003, p. 188).

31 Even if that means that the range of beliefs such institutions are capable of forming is to be restricted only to those relevant to the fulfillment of these goals (Pettit, 2003, p. 188).

32 It’s worth noticing that our account in principle allows for the idea that institutions could lie to individuals or other institutions (as in Sorensen’s example in which the British Navy lied to the German Navy about magnetic mines sinking ships (Sorensen, 2022, p. 130,7)) as according to some theorists, they are capable of asserting and intending to deceive (see Lackey, 2018; Marsili, 2023b).

33 Dynel (2011) argues that bald-faced lies are not lies because they are overtly untrue. We agree that those falsehoods that are overtly untrue to all agents involved are not lies, but we think that in typical cases of bald-faced lies the falsehoods uttered are not overtly untrue to all agents: the institutions involved accept those falsehoods as true (i.e. they put them on the record).

34 As did one of the anonymous referees for this journal.

35 Sorensen considers many different examples, each of which is worthy of a separate discussion, for which, alas, we have no space here.

36 Sorensen claims that the boy’s utterance is “a bald-faced lie with respect to his sister”. We disagree. If the sister is in on the lie and knows that her twin brother says this only to deceive their younger brother, he doesn’t lie to her at all. From her perspective his utterance is not a proper assertion, it is a kind of pretense. Thus, we would say that although the boy lies, he doesn’t lie to his sister. Scaling up has the consequence that an utterance might be a lie and yet not be a lie to a particular hearer.
